# Chicago Medical Society, Meeting of Jan. 20

**Published:** 1879-02

**Authors:** R. Park


### ARTICLE IX.

CHICAGO MEDICAL SOCIETY. REGULAR MEETING, JAN. 20.
Reported by R. PARK, M. D.

DR. W. H. BYFORD repeated most of a clinical lecture (which is printed in this number of the JOURNAL AND EXAMINER) on "Puerperal Vaginitis, as a Cause of Vesico-vaginal Fistula."

In the discussion which followed, Dr. Dudley said he had seen cases, and made autopsies, where urine passed through fistulæ which were so small or contracted that they escaped detection. The plan of closing the vulva when most of the posterior wall of the bladder was gone, or when the fistula was too large to be closed, seemed to him an excellent one, inasmuch as a large receptacle was thus formed for urine.

DR. RARTLETT said he had once attended a woman in labor who had a prolapsus of the bladder. He introduced a flexible catheter, evacuated and replaced the viscus, and delivered her naturally and safely. He afterwards learned that she had suffered from cystocele for several years.

DR. FLOOD thought that forceps themselves might cause injury which would lead to fistula in a more direct way than by causing puerperal vaginitis.

DR. INGALS asked Dr. Byford whether the danger was great when the pressure, caused by the child's head, was severe, though of short duration.

In closing the discussion Dr. Byford claimed that the pressure of the head on the soft parts did not cause nearly as much damage *directly* as did the inflammation thus *indirectly* excited. Not enough attention had been directed to this point. Treatment *after* labor was much more frequently at fault than treatment *during* labor. He had labored hard to make this matter better and more generally understood. In answer to Dr. Flood's question, he thought it next to impossible, under natural conditions, to rupture the bladder. The vaginal walls were tightly stretched about the head, and were very strong, and he could hardly conceive how the walls could be caught. But, of course, an ignoramus could produce almost any injury—even punching a hole through the bladder. He had once cured a case of fistula, and was subsequently called to attend the same woman in labor. He delivered with forceps, knowing full well the danger, and was unable to prevent a fresh laceration and rupture of the septum. But he had the satisfaction of saving the child and afterwards of again curing the mother. To Dr. Ingals' question, he replied that prolonged pressure might cause danger. He thought that when the head seemed impacted and neither advanced nor receded, forceps should be used. He had seen one case where a severe laceration or rupture had been produced by the bungling use of the perforator.

As germane to the subject, Dr. Etheridge exhibited a Smith's cleft-palate needle, of English make, which he considered equally well adapted for closing fistulæ. A hollow needle was employed and the suture wire passed through it.

DR. BYFORD exhibited a tumor nucleated from the vesico-vaginal septum, by the descent of the fœtal head. The tumor had bony walls of true cancellous tissue, and on opening it there were found hair, irregular, small bones, and sebaceous matter. Its longest diameter was about 10 cm. He considered it a dermoid cyst which had developed in the anterior vaginal wall.

DR. C. T. FENN reported the autopsy of a case of gall stone, which caused an abscess, with destructive changes in the vicinity of the gall bladder ; the abscess finally breaking into the pylorus.

DR. HAYES also showed a large gall stone, weighing 15-6 grams, taken from a patient that died of shock following ovariotomy. She had never complained of anything to excite suspicion more than pain in her right side.

